# Wealth and under-nourishment among married women in two impoverished nations: evidence from Burkina Faso and Congo Democratic Republic

**DOI:** 10.1186/s13104-015-1001-7

**Published:** 2015-02-08

**Authors:** Ayo Stephen Adebowale, Martin Enoch Palamuleni, Clifford Obby Odimegwu

**Affiliations:** Population Training and Research Unit, Faculty of Humanities and Social Sciences, North-West University, Mafikeng, South Africa; Department of Epidemiology and Medical Statistics, Faculty of Public Health, College of Medicine, University of Ibadan, Ibadan, Nigeria; Department of Population studies and Demography, University of Witswatersrand, Johannesburg, South Africa

**Keywords:** Poor nutritional status, Married or cohabiting women, Poverty, Body mass index

## Abstract

**Background:**

Burkina Faso (BF) and Congo Democratic Republic (CDR) are among the top-ten poverty and hunger stricken countries globally. The influence of poverty and hunger on health is enormous. The objectives of the study are to; examine the association between poverty and nutritional status, it also identified socio-demographic and health related mediating factors that contribute to the relationship between poverty and poor nutritional status. The study focused on married or cohabiting women aged 15–49 years and utilized 2010 and 2007 DHS dataset from BF and CDR respectively.

**Findings:**

Mean age of the women in BF and CDR were 34.4 ± 9.3 and 34.7 ± 9.0 years respectively. About 19.4% and 18.4% of the poor were malnourished as against 7.7% and 9.7% of the rich women in BF and CDR respectively. Obesity and overweight were more prominent among the rich than the poor. Higher prevalence of under-nourish women was found among the older than the younger women in BF. In the countries, the prevalence of malnutrition was significantly higher among women; in the rural areas, with no formal education, anaemic and those who are not working. Multivariate analysis revealed that in the countries, the risk of under-nourishment was significantly higher among poor and middle class than the rich women despite controlling for confounding variables.

**Conclusions:**

Undernourished women were more common among the poor and those with no formal education. Programs that target nutrition of women of reproductive age should be strengthened in BF and CDR.

## Background

Poverty is associated with the worsening of a range of key human attributes, including health. The poor are exposed to greater health risks, are limited in accessing nutritious food and health care. Consequently, they have a higher risk of illness and disability [1]. Illness resulted from poverty can reduce household savings, lower learning ability, reduce productivity, and lead to a poor quality of life, thereby escalating poverty [1]. In every society, there is a socially acceptable minimum level of living of which individuals who live below is said to live in poverty. Thus, poverty is a condition in which there is dearth of indispensable facilities or failure to meet basic needs [1,2]. Objectively, one can argue that poverty does not merely mean lack of adequate income or inability to meet basic daily needs. This is because some people do have the potential to get themselves out of poverty; they have good health and can live a productive life but still deprived of suitable opportunities [2]. The implied denial of opportunities pushes them into unemployment and makes it impossible for them to meet their basic necessities of life [2].

Poverty is found in any society and is a vast social phenomenon; however, its severity differs between the developed and developing countries. Differential in the pace of socioeconomic growth is comparatively high in the developed countries, leading to a wider gap between the standard of living of their citizens and that of developing countries. Due to poverty, most inhabitants in poor countries are unable to meet their daily dietary needs and as such their health and nutrition are compromised. Previous studies have linked poverty to malnutrition [3-5].

Globally, malnutrition constitutes public health challenges. The health implication of malnutrition is colossal and its consequences on women particularly during child bearing and rearing years cannot be over-emphasized [6,7]. For instance; anaemia during pregnancy, lost of blood at delivery, pre-term birth, low birth weight, lost of quantum amount of calorie during breastfeeding and emotional care and support for young children and entire family make women of reproductive age more susceptible to poor nutrition than any other segment of the population [6,8]. However, the effect of these health indices could be curtailed if pregnant women have the financial capacity to access balanced diet and adequate dietary intake. In sub-Saharan Africa region, poverty and hunger ravage the population and majority of the people resident in the region live below poverty line [9-11]. This is evidenced in the number of countries from the region on the list of top-ten poor and hunger affected countries globally among which Burkina Faso and Congo Democratic Republic from the west and central Africa respectively are included [10,12]. The two countries are francophone.

In Burkina Faso and Congo Democratic Republic as for most countries in sub-Saharan Africa, food substances that give calories needed for energy, growth and survival are not within the reach of poor. Regrettably, some poor households who engaged in subsistence farming often sell their farm produce in order to meet other daily needs [13]. As an example, a study conducted in Ghana revealed that the majority of the farming households were food insecure [14], thus, compromising the health of young members of the family and women who are the care provider for the family. The attitude of Governments of countries in sub-Saharan Africa with respect to peoples’ welfare and well-being is not helping the matter. Socioeconomic infrastructures that can enhance the quality of life of the poor are lacking and the existing structures are already collapsing. People are unemployed and deprived opportunities, the leaders drain off public fund to the detriment of the citizens, thus creating a gap between the rich and the poor. Green opined that to be deprived of opportunities and being unemployed is to remain in poverty and lack of opportunity in economic and political life is one of the root causes of poverty [2]. Inequalities perpetuated in political, economic and social systems can cause poverty among certain segments of the population due to systems that make it difficult for that segment to thrive [15]. Thus the poor which have little or no means of affording foods that contain essential nutrients for their growth and survival are always at the receiving end.

Micronutrient deficiencies can; impair individual cognitive ability, abate individual immune systems, increase the level of mortality, and hinder individual productivity are barriers to socioeconomic development and growth of a nation [16]. Women of low socioeconomic status are most susceptible to food insecurity due to their poor purchasing power. Households that cannot afford nutritious foods due to low income are mostly linked with the insufficient diet and disease that leads to undernourishment. Such households usually spend the bulk of their total income on food. Any health problem that requires spending part of the meagre family income can further perpetuates food insecurity [17]. The associated health consequences of malnutrition in early years of life may forever put at risk the health of such children [18]. For instance, infant under-nutrition has been found to be associated with shorter adult stature, promotes absenteeism, less schooling achieved and compromises socioeconomic advancement in life [18].

HIV/AIDS, is known to be mostly common among women of childbearing age than any other age segment of the population [19]. A serious challenge to the nutrition of women in sub-Saharan Africa in contemporary times is HIV/AIDS, which has been of greatest risk to poor populations than the rich [20]. The rich living with the disease can have a better financial ability to cope with the health effect of the disease by eating nutritious food and having better access to ARV drugs unlike their poor counterparts. Although, the prevalence of HIV/AIDS among women of reproductive age in Burkina Faso (1.5%) and Congo Democratic Republic (1.6%) are not as high as other countries in sub-Saharan Africa, but the thousands of women affected in these countries are susceptible to under-nutrition if they have poor access to nutritious food [19].

The first and fourth indicators of the Millennium Development Goal indicators to eradicate extreme poverty and hunger and improve maternal health [21] emphasize the need to explore the relationship between the state of nutrition of women and poverty in Burkina Faso and Congo Democratic Republic as evidenced in this study. Our study examined the role of poverty on nutritional status of married or cohabiting women against the backdrop of dearth of research on this subject matter in these countries. The objectives of the study are to; examine the association between wealth index and nutritional status, it also identified socio-demographic and health related mediating factors that contribute to the relationship between wealth index and undernourishment. This is with the view to ascertaining the importance of wealth as key determinant of being undernourished in the studied countries.

## Methods

### Background information of the studied countries

Burkina Faso and Congo Democratic Republic are among the top ten World’s hunger and malnutrition countries. Burkina Faso has a population figure of 17.5 million and TFR of 6.0 children per woman. The birth rate is 43 per 1,000 populations, female life expectancy is 56 and death due to non communicable diseases is 713 per 100,000 population [19]. The background information of Congo Democratic Republic is similar with 69.7 million inhabitants and TFR of 6.3 children per woman. The birth rate is 45 per 1,000 populations, female life expectancy is 50 and death due to non communicable diseases is 806 per 100,000 population [19]. The per capita GDP growth rate in Burkina Faso and Congo Democratic Republic was 4.9% and 5.3% respectively. Also, the percentage shares of income for the two countries are Burkina Faso {Poorest (7) and Wealthiest (47)} and Congo Democratic Republic {Poorest (5) and Wealthiest (51)} [19].

### Data collection

The study focused on married or cohabiting women of reproductive age (aged 15-49years) from the most recent rounds of Demographic and Health Surveys (DHS) in Burkina Faso and Congo Democratic Republic [22,23]. The selected DHS are: Burkina Faso, 2010 and Congo DR, 2007. The selection was based on data availability at meeting the goals of the study and the two countries share some similar background information associated with poverty and poor nutrition.

### Study sample

We extracted the data from the measure DHS database and as such, the methodologies involved in the collection process are available to interested readers in the DHS reports of the selected countries obtainable from measure DHS website (http://www.measuredhs.com/). In this study, a sub-Sample which comprises of married or cohabiting women aged 15–49 years was selected across the two countries for the analysis. In Burkina Faso and Congo Democratic Republic, the sub-Samples were 2939 and 1294 women respectively. Women who were either pregnant or breastfeeding were excluded from the study. For, pregnant women, the weight of the foetus could not be determined during the survey so as to deduct it from the measured weight of the women. Also, lactating mothers were excluded because they are likely to lose a lot of calories during breastfeeding period particularly those who are practicing exclusive breastfeeding.

Other set of women excluded includes, women who were either singles, widows or separated. Also, these set of women are less exposed to fertility which has significant influence on nutritional status of women.

### Dependent variable

The dependent variable was nutritional status (Body Mass Index (BMI)) and this was generated using the information on height and mass of the respondents. Body Mass Index (BMI) is a simple index of weight-for-height that is commonly used to classify underweight, overweight and obesity in adults. *BMI = Mass* / (*height*)^2^ where; mass was measured in kilogramme and height in metre. The resulting variable was categorized into four as; under-nourish if *BMI < 18.5*; Normal if *18.5 ≤ BMI < 24.99*; Overweight if *25 ≤ BMI < 29.99* and Obesity if *BMI ≥ 30*. The classification was based on WHO standard guideline for measuring Adult nutritional status [22,23].

### Independent variable

The key independent variable is wealth index which was used as a measure of poverty. For the computation of wealth index, principal components analysis (PCA) was used to assign the wealth indicator weights. This procedure firstly assigned scores and standardized the wealth indicator variables such as; bicycle, cars, building type, e.t.c. Thereafter, the factor coefficient scores (factor loadings) and z-scores were calculated. Finally, for each household, the indicator values were multiplied by the loadings and summed to produce the household’s wealth index value. The standardized z-score was used to disentangle the overall assigned scores to poorest, poorer, middle, richer, richest categories.

However, in our study, the variable was further collapsed into three categories by recoding poorest and poorer as poor; richer and richest as rich; while the middle class remain as being intermediate indicator between the rich and the poor. The re-classification was based on African context where an individual is seen as either rich or poor or belonging to middle class. Other variables included are age, residence, having birth in the last 3 years, children ever born, education, husbands’ education, work status of the woman, anaemia and marital status.

### Data analysis

The dependent variable was re-coded into two categories as; $$ \mathrm{B}\mathrm{M}\mathrm{I} = \left\{\begin{array}{c}\hfill 1\  if\ BMI\ \le\ 18.0\hfill \\ {}\hfill 0\  if\  otherwise\hfill \end{array}\right. $$Where; 1 is an indicator of malnourish or poor nutrition. The WHO regards a BMI of less than 18.5 as underweight and may indicate malnutrition, an eating disorder, or other health problems. Logistic regression was used to examine the relationship between poor nutrition and poverty status (wealth index). Other socio-demographic and health related variables were also included to see their influence on the relationship between poor nutrition and poverty status.

### Ethical approval

Prior the survey, ethical approval was obtained by the data originator (Measure DHS) from National Ethics Committee of the countries involved in this study. An informed consent was obtained from all the study participants after describing to them all the issues related to the study in details at the point of data collection. Eligible respondents who did not want to partake in the study were excluded from the survey. Each consented participants was made to sign appropriate agreement form before the interview. However, we requested for the permission to use the data for this study online via DHS web-platform and formal approval was granted by measure DHS before the commencement of this study. All the women that participated in the study were married as at the time of the study and as such there was no need for young women under 16 years to receive informed parental consent for their participation.

## Results

**Figure 1:**
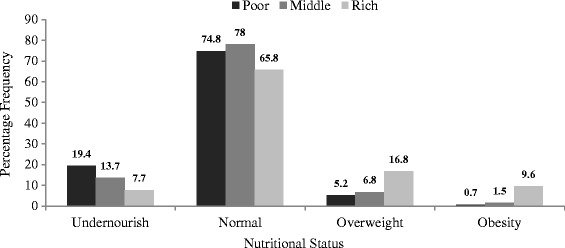
**Nutritional status of married or cohabiting women in Burkina Faso by wealth index.**

In Figure 1, the data show that among married or cohabiting women in Burkina Faso, 19.4% and 7.7% of the poor and rich women were under-nourished respectively. The percentage of rich women that had normal nutritional status was the least (65.8%) compared to women who were poor (74.8%) and in the middle class (78.0%). The prevalence of obesity and overweight are more prominent among the rich women than either poor or middle class women.

**Figure 2:**
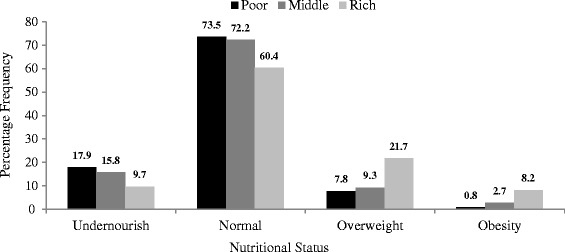
**Nutritional status of married or cohabiting women in Congo Democratic Republic by wealth index.**

In Congo Democratic Republic, the pattern of nutritional status of the women studied was similar to that of Burkina Faso except that the prevalence of women with normal nutritional status was highest among the poor (73.5%). About 18% of the poor were under-nourished as against 9.7% of the rich women. Consistently, the level of overweight and obesity increases with increasing in the level of wealth quintile as shown in Figure 2.

**Figure 3:**
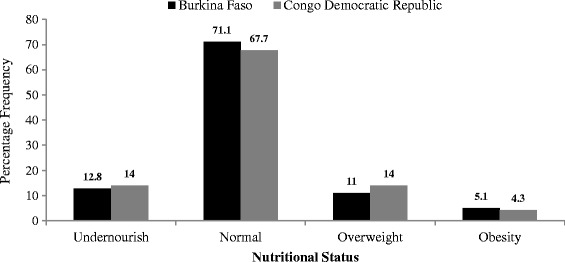
**Nutritional status of all married or cohabiting women in Burkina Faso and Congo Democratic Republic.**

Among all the married or cohabiting women in the studied countries, the prevalence of under-nourished women was higher in Congo Democratic Republic (14.0%) than Burkina Faso (12.8%). Whereas 71.1% of women in Burkina Faso had normal nutritional status, lower percentage of these women was found in Congo Democratic Republic (67.7%). Obesity was more common among women in Burkina Faso (5.1%) than those in Congo Democratic Republic (4.3%). Comparing the nutritional status shows that similar pattern and slight discrepancy exists between the countries as shown in Figure 3.

Mean age of the women in Burkina Faso and Congo Democratic Republic were 34.4±9.3 and 34.7±9.0 respectively. In both countries, the mean age of under-nourished women was higher than the overall figure in each of the countries. In Table 1, the data show that in the two countries, there was significant association between wealth index and nutritional status of the women. While age and children ever born were significantly associated with nutritional status in Burkina Faso, these variables were found to be insignificant in terms of their association with nutritional status in Congo Democratic Republic. There was an evidence of higher prevalence of under-nourishment among the older women than the younger women in Burkina Faso. Although, similar pattern was observed in Congo Democratic Republic but age was found to be insignificantly associated with nutritional status. The percentage of under-nourish women was significantly higher among women who either had no birth (12.9%) or already given birth to at least five children (15.8%) than those who gave birth to 1–2 (8.4%) and 3–4 (11.0%) children.

**Table 1:** **Percentage distribution of married or cohabiting women in Burkina Faso and Congo Democratic Republic according to level of poor nutritional status by background characteristics**

**Background characteristics**	**Burkina Faso**			**Congo Democratic Republic**		
	**Under nourished**	**Total women**	**χ** ^**2**^ **-value (p-value)**	**Under nourished**	**Total women**	**χ** ^**2**^ **-value (p-value)**
Total	12.8(376)	2939		14.0(181)	1294	
**Key independent variable**						
Poverty status			72.039***			15.300***
Poor	19.4(195)	1006	(p<0.001)	17.9(87)	487	(p<0.001)
Middle	13.7(73)	532		15.8(41)	259	
Rich	7.7(108)	1401		9.7(53)	548	
**Demographic variables**						
Age			23.511***			2.227
15-24	12.6(68)	540	(p<0.001)	14.8(31)	209	(0.527)
25-29	6.3(26)	413		13.7(28)	205	
30-39	12.5(117)	933		12.1(50)	414	
40-49	15.7(165)	1053		15.5(72)	466	
Mean±σ	35.8±9.7	34.4±9.3		35.3±9.5	34.7±9.0	
Children ever born			24.049***			0.046
None	12.9(40)	311	(p<0.001)	14.1(23)	163	(0.997)
1-2	8.4(53)	633		14.1(43)	305	
3-4	11.0(74)	675		13.6(40)	294	
5+	15.8(209)	1320		14.1(75)	532	
Mean±σ	4.8±2.9	4.2±2.9		4.0±2.9	4.1±3.0	
Births in the last 3 years			0.045			0.228
None	12.9(283)	2199	(0.832)	14.3(134)	939	(0.633)
1+	12.6(93)	740		13.2(47)	355	
**Socioeconomic variables**						
Residence			59.703***			5.887*
Urban	5.9(55)	940	(p<0.001)	11.4(68)	594	(0.015)
Rural	16.1(321)	1999		16.1(113)	700	
Education			40.405***			8.608*
None	14.9(341)	2295	(p<0.001)	18.1(56)	309	(0.014)
Primary	6.1(23)	376		14.5(73)	503	
Secondary+	4.5(12)	268		10.8(52)	482	
Husband’s education			42.460***			15.570***
None	15.0(331)	2206	(p<0.001)	24.4(30)	123	(p<0.001)
Primary	4.9(18)	366		15.8(52)	329	
Secondary+	6.5(22)	341		11.4(90)	791	
Work status			5.882*			3.777
Not working	16.2(80)	494	(0.015)	10.5(30)	286	(0.052)
Working	12.2(293)	2405		15.0(151)	1006	
Marital status			5.549*			0.770
Married	13.1(366)	2787	(0.018)	13.6(147)	1080	(0.380)
Cohabiting	6.6(10)	152		15.9(34)	214	
**Health related variable**						
Anaemia status			8.920**			5.298*
Not anaemic	11.1(172)	1550	(0.003)	11.8(75)	635	(0.021)
Anaemic	14.8(200)	1350		16.3(100)	612	

***Significant at 0.1%; **Significant at 1%;*Significant at 5%.

All the socioeconomic factors such as; residence, education, work status, marital status and husband’s education used in this study were found to be significantly associated with nutritional status in Burkina Faso but only residence, education and husband’s education were found in Congo Democratic Republic. Higher proportion of women in urban area of Congo Democratic Republic (11.4%) was under-nourished than the urban area in Burkina Faso (5.9%) but in the rural areas of the two countries the prevalence of under-nourish women were the same (16.1%). The percentage of women who were under-nourished reduces consistently with increasing level of education of the women and that of their husband. This pattern is similar for the two countries investigated. It is striking, that while 14.9% and 18.1% of women with no formal education were undernourished in Burkina Faso and Congo Democratic Republic respectively, fewer proportion 4.5% and 10.8% were found among women with at least secondary education in the two countries. It is also interesting to know that in Burkina Faso, women who engaged herself in one work activity or the other had lower proportion of their members (12.2%) under-nourished than those who were not working (16.2%). Also, differential exists in poor nutritional status of married (13.1%) and cohabiting (6.6%) women in Burkina Faso. As expected, across the countries, higher proportion of women who were anaemic at the time of the survey was found to be underweight than those who were not.

### Multivariate analysis

Table 2 presents the multivariate output of this study. In the multivariate analysis, 5 models were generated for each country to explore the influence of the independent variables on the nutritional status. The first model involved only wealth index, whereas the second included the demographic variables. The third and the fourth models included the socioeconomic and health related characteristics respectively while all the variables were used as control in the fifth model. Across all the models, the data show that the risk of under-nourished was significantly higher among poor and middle class women than the rich women and the pattern is similar for the two countries. For instance, among women in Burkina Faso and Congo Democratic Republic those who were classified as being poor were 2.88(p<0.001) and 2.03(p<0.001) times respectively more likely to be under-nourished than the rich women. While controlling for possible confounding variables, the ORs declined quite substantially, from 2.9 to 1.7, in Burkina, for instance. This suggests that in the country, part of the odds associated with poverty is explained by age, residence, husband’s education, working status and anaemia status.

**Table 2:** **Logistic regression models of the relationship between poverty status and under-nourish among married or cohabiting women in Burkina Faso (BF) and Congo Democratic Republic**

**Background variables**	**Model 1**		**Model 2**		**Model 3**		**Model 4**		**Model 5**	
	**BUF**	**CDR**	**BUF**	**CDR**	**BUF**	**CDR**	**BUF**	**CDR**	**BUF**	**CDR**
**Key independent variable**										
*Poverty status*										
Poor	2.9***	2.0***	2.6***		1.8***	1.8*	2.8***	1.9***	1.7***	1.6
Middle	1.9***	1.8***	1.8***		1.2	1.8*	1.8***	1.8**	1.2	1.8*
Rich *(Ref. C)*	1.0	1.0	1.0		1.0	1.0	1.0	1.0	1.0	1.0
**Demographic variables**										
*Age*										
15-24			1.1						0.9	
25-29			0.5*						0.5*	
30-39			0.9						0.9	
40-49*(Ref. C)*			1.0						1.0	
*Children ever born*										
None			0.8						0.9	
1-2			0.6*						0.7	
3-4			0.9						0.9	
5+ *(Ref. C)*			1.0						1.0	
**Socioeconomic variables**										
*Residence*										
Urban					0.5**	1.3			0.5**	1.2
Rural *(Ref. C)*					1.0	1.0			1.0	1.0
*Education*										
None					1.9	1.1			1.7	1.1
Primary					1.1	0.9			1.0	0.9
Secondary+ *(Ref. C)*					1.0				1.0	1.0
*Husband’s education*										
None					1.2	2.1**			0.9	2.3**
Primary					0.4*	1.3			0.4*	1.3
Secondary+ *(Ref. C)*					1.0	1.0			1.0	1.0
*Work status*										
Not working					1.6**				1.6**	
Working *(Ref. C)*					1.0				1.0	
*Marital status*										
Married					0.9				0.9	
Cohabiting *(Ref. C)*					1.0				1.0	
**Health related variable**										
*Anaemia status*										
Not anaemic							0.8*	0.7*	0.8	0.7
Anaemic *(Ref. C)*							1.0	1.0	1.0	1.0
**−2logLL**	**2175.9**	**1031.7**	**2154.2**		**2080.4**	**977.4**	**2145.6**	**992.5**	**2039.7**	**937.6**

***Significant at 0.1%; **Significant at 1%; *Significant at 5%; BUF: Burkina Faso; CDR: Congo Democratic Republic.

The data further show that, in Burkina Faso and Congo Democratic Republic, the likelihood of being under-nourished was a half lower among women aged 25–29 than those between the ages of 40 and 49 years. Women in rural areas were twice more likely to be under-nourished than those in urban areas. Also, the data show that women who are not anaemic are at lower odds of under-nutrition. In Congo Democratic Republic, the likelihood of under-nourished women was found to be higher among women whose their husbands had no formal education than those with at least secondary education. But, in Burkina Faso, the risk of under-nourishment was found to be lower among women with primary education than those with at least secondary education. In Burkina Faso, the risk of under-nourishment was found to be significantly higher among women who were not working than their counterparts who were working (models 3 and 5).

## Discussion and conclusions

The Millennium Development Goal 5 to reduce maternal mortality by three-quarters in 2015 targets nutritional status of women. Poor maternal nutrition may be linked with women’s lack of resistance to ill health during pregnancy, childbirth and childrearing. Poor financial ability to intake of nutritious food substances by women at different stages of reproductive life constitutes a serious challenge to maternal health. Under-nourishment reduces the pace of social and economic development of an individual, community and nation. As part of measures to designing appropriate interventions to reduce the prevalence of malnutrition among women, it is quite necessary to know how many women are under-nourished and identify socio-demographic factors responsible for the menace. Malnutrition is the result of marginal dietary intake compounded by infection. While poverty is not the sole determinant of nutritional status of a woman, this study is evidenced that it is an important factor to reckon with in impoverished nations like Burkina Faso and Congo Democratic Republic.

We found that the prevalence of malnutrition was higher among the poor than the rich in the two countries, however, being overweight or obese was common among the rich women than either the poor or middle class women as previously found in a study conducted in Nigeria [24]. Poor socioeconomic status of the majority of the people in the studied countries could be a possible explanation for our finding [12]. However, in contrary, trend analysis conducted by different researchers in different settings found that nutritional status was better among adults in the lowest income group than those who belong to the highest income category [25-28].

Our study also revealed that the prevalence of acute under-nourishment among women was higher in Congo Democratic Republic than Burkina Faso while higher proportion of women in Burkina Faso had normal nutritional status, overweight and obese than their counterparts in Democratic Republic. The pattern of differential could be attributed to striking differences in population of people living below the poverty line in the two countries with the higher figure found in Congo Demographic Republic [12]. Under-nourishment has long been recognized as a consequence of poverty. It is widely known that increasing rates of under-nourished women are found in areas with chronic widespread poverty [29]. For instance, the World data bank reveals that 71.3% and 46.7% of people in Congo Democratic Republic and Burkina Faso live below the national poverty line respectively [12]. Also, higher proportion of the population in Congo Democratic Republic (87.72%) in 2006 earn less than $1.25 dollar per day as against 44.6% in Burkina Faso in 2009 [12].

The prevalence of under-nourished women was higher among older women in Burkina Faso than the younger women. This finding is consistent with the study conducted by Forster and colleague where an increasing age was found to be independently associated with undernutrition [30]. However, in a similar survey that was conducted in Bangladesh, the study’s outcome was at variance with our finding [31]. In Burkina Faso and Congo Democratic Republic, under-nourishment was found to be higher among women who were either yet to bear any children or already had more than five children than those who had between 1 and 4 children. This finding corroborates the result of a study in Nigeria where prevalence of under-nourishment was found to be higher among women with higher parity [32].

We further found that under-nourishment was strikingly more prominent among women in the rural than those in the urban areas in Burkina Faso and Congo Democratic Republic. This is in line with the DHS conducted in Ethiopia and a similar study in Bangladesh [33,34]. Although, the proportion of under-nourished women in urban area of Burkina Faso was lower than that of their counterparts in urban areas of Congo Democratic Republic, there was no difference in the level of under-nourishment in the rural areas of the two countries.

There was consistent reduction in the proportion of under-nourished women as level of education of the women and that of their husband increases. Earlier studies conducted in Nigeria and India observed similar patterns [32,35]. The pattern observed in this study may be due to the fact that more educated women are expected to be involved in job or work activities that earn better income. Also, ability to rationalize household food items and having varieties even when it was not adequately available may be more common among the highly educated elites than their counterparts with less education.

In Burkina Faso, employment status was significantly associated with nutritional status. It was found that the proportion of employed women who were under-nourished was lower than that of those who were not working. This is in agreement with the study carried out in Ethiopia where women’s employment status was found to be an important determinant of nutritional status among women of reproductive age [36]. These authors argued that this may be due to women’s economic influence within the household through their participation in income-generating activity. The reverse pattern was found in Congo Democratic Republic but the association was not significant. In the analysed countries, being anaemic predisposes women to malnutrition.

The multivariate analysis revealed that wealth index was significantly related to nutritional status of women. We found that the likelihood of under-nourishment was higher among the poor than the rich in both Burkina Faso and Congo Democratic Republic. The strength of the relationship remains unchanged when other variables were used as control. In Burkina Faso, aside wealth index, variables such as age, residence, husband’s education, work status were identified as the predictors of under-nourishment whereas wealth index and husband’s education were the important predictors of under-nourishment in Congo Democratic Republic.

In conclusion, the interrelationship between wealth index and nutritional status as found in this study demonstrates that poverty is a major socioeconomic determinant of under-nourishment among women of reproductive age in Congo Democratic Republic and Burkina Faso. Under-nourished women were more common among the poor and those with no formal education.

### Implication for practice

Programs that target nutrition of women of reproductive age should be strengthened in Congo Democratic Republic and Burkina Faso. Government should also develop effective intervention programmes aim at improving people’s nutritional status. To gain a better insight into the underlying cultural mechanisms necessary for the attainment of adequate nutrition, further research using qualitative methodology is required.
